# ESPriT1: the effectiveness of laparoscopic treatment of isolated superficial peritoneal endometriosis for managing chronic pelvic pain in women: a randomised controlled feasibility trial

**DOI:** 10.1530/RAF-26-0043

**Published:** 2026-06-01

**Authors:** Lucy H R Whitaker, Linda J Williams, Jacqueline Stephen, Jane P Daniels, Andrew W Horne

**Affiliations:** ^1^ Centre for Reproductive Health, University of Edinburgh, Edinburgh, UK; ^2^ Edinburgh Clinical Trials Unit, Usher Institute, University of Edinburgh, Edinburgh, UK; ^3^ Nottingham Clinical Trials Unit, University of Nottingham, Nottingham, UK

**Keywords:** feasibility RCT, superficial peritoneal endometriosis, chronic pelvic pain, endometriosis, laparoscopy

## Abstract

Endometriosis is a chronic pain condition that affects >190 million people worldwide. It is recommended in clinical guidelines to remove the most common type of endometriosis, ‘superficial peritoneal endometriosis’ (SPE), if it is diagnosed at laparoscopy (keyhole surgery). However, the evidence to support removal of SPE is based on small studies with short-term follow-up. The aim of this small feasibility trial was to determine what proportion of women with pelvic pain undergoing laparoscopy for suspected endometriosis would be willing to be randomly allocated to have surgical removal of SPE or not have it treated surgically straightaway (sham surgery). Of the eligible patients that were approached, 31% consented to take part, demonstrating that people with endometriosis are willing to take part in a sham surgical trial. We are now leading a similar, but much larger UK-wide trial, to determine whether removal of SPE truly improves painful endometriosis symptoms.

Superficial peritoneal endometriosis (SPE) is the most common endometriosis subtype and can be associated with debilitating pelvic pain (Horne *et al.* 2019). Laparoscopic removal of SPE lesions, by excision or ablation, is frequently performed to alleviate symptoms, but existing clinical trials to support this treatment approach are underpowered, have short-term follow-up and typically include other endometriosis subtypes (Horne *et al.* 2019, Bafort *et al.* 2020). International endometriosis guidelines recognise that better-quality evidence is required (Becker *et al.* 2022).

The aim of this feasibility trial, ESPriT1, was to determine what proportion of women undergoing diagnostic laparoscopy for suspected SPE would agree to be randomly allocated to diagnostic laparoscopy or concurrent surgical removal of disease, to inform the design of a future large UK trial. In the definitive trial, our objective is to determine whether surgical removal of SPE improves overall pain symptoms and quality of life or whether surgery is of no benefit or exacerbates symptoms. The use of a diagnostic laparoscopy alone as the appropriate comparator in order to determine efficacy of surgical removal was supported by UK surveys of clinicians and patients, and our study patient advisory group, and approved by the reviewing ethics committee. The trial registration number is NCT04081532.

Women with chronic pelvic pain and suspected SPE listed for diagnostic laparoscopy were recruited over a 16-month recruitment period from outpatient clinics in three UK centres. At the time of surgery, if SPE in isolation was observed, they were randomised 1:1 to diagnostic laparoscopy alone or surgical removal of all visible endometriosis (Whitaker *et al.* 2021). Participants were blinded to treatment allocation. Participants were followed up by online questionnaires at three, six and 12 months following surgery to assess pain, physical and emotional function. An acceptability questionnaire was completed at 12 months. The primary outcome was the proportion of eligible participants who agreed to randomisation.

Between October 2019 and February 2021, 57 of 181 eligible patients consented to the trial (31%, 95% confidence interval (CI): 25–39%). Of these, 26 underwent surgery during this period, of which seven were randomised (see Fig. 1). The main reason (78.9%) for non-randomisation was that no pelvic pathology was observed at the time of surgery. The COVID pandemic considerably impacted randomisation following consent because elective gynaecological surgery was paused for six months during the recruitment phase, and on resumption, surgical waiting-lists were substantially increased. Consequently, 28/57 consented patients (49%) had not yet undergone surgery by the end of the study.

**Figure 1:**
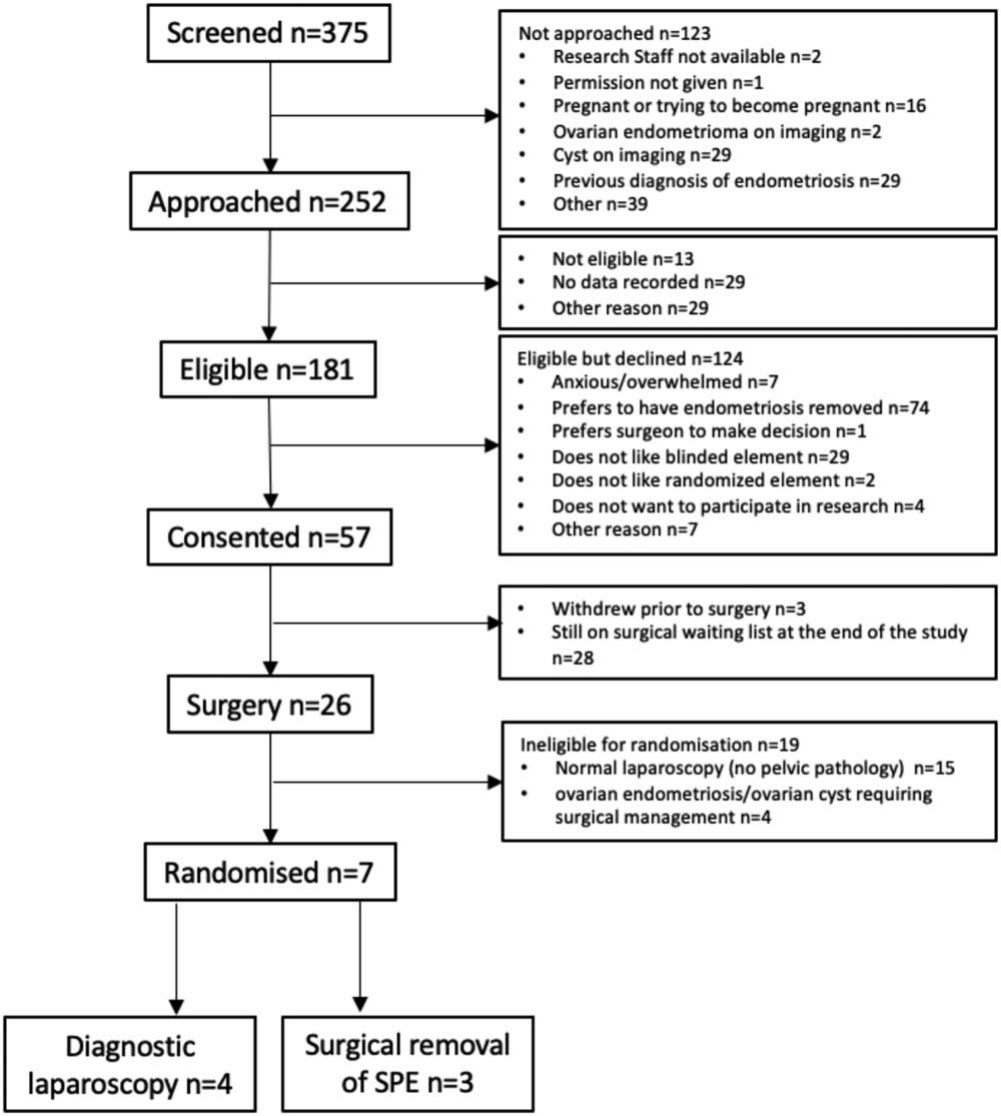
CONSORT diagram.

The upper 95% CI of recruitment rate was 39%, below the pre-specified 40% consent rate of eligible participants that was considered acceptable to proceed to a definitive trial. However, this shortfall likely reflects the substantial disruptions caused by the COVID-19 pandemic. The trial exceeded its pre-specified retention rate, and all of the randomised participants returned 12-month outcome data and remained blinded to study end. Further secondary outcome data are reported in the Supplementary materials (see section on Supplementary materials given at the end of the article). Consequently, we concluded that our design supported retention and, with sufficient recruiting sites, a definitive trial was feasible.

This study has informed the design and funding for our current multicentre trial ESPriT2 (Mackenzie *et al.* 2023) to determine the efficacy of surgical removal of SPE for endometriosis pain. Ultimately, the findings will empower those affected by endometriosis to make evidence-based decisions regarding surgical treatment, while also informing the development of endometriosis care infrastructure – whether through enhanced access to surgical interventions or through the advancement to symptom management.

## Supplementary materials



## Declaration of interest

LHRW received grant funding from the NIHR, UKRI, Chief Scientist’s Office and Wellbeing of Women. LHRW’s institution received grant funding from Roche Diagnostics and honoraria for teaching from Gedeon Richter and Theramex. JPD received grant funding from the NIHR, Chief Scientist’s Office and Wellbeing of Women. AWH received grant funding from the NIHR, MRC, Chief Scientist’s Office, Wellcome Trust, Wellbeing of Women, Ferring and Roche. AWH’s institution received honoraria for consultancy from Roche Diagnostics, Gesynta and Joii; grant funding from Roche Diagnostics; and honoraria for teaching from Gedeon Richter. AWH has received lecture fees from Theramex. The other authors declare no competing interests. A W Horne is co-Editor-in-Chief of *Reproduction & Fertility* and was not involved in the review or editorial process for this paper, on which he is listed as an author.

## Funding

This research was funded by MRC (Grant No. MR/N022556/1) and the Chief Scientist Office (Award No. TCS/18/43).

## Author contribution statement

All authors contributed to the conception, design and delivery of the study. AWH and JPD secured funding. AWH, LHRW, JPD, JS and LJW were the trial management group. LHRW was PI for the lead site. JS and LJW performed the statistical analyses. LHRW, LJW, JS and AWH drafted the report, and all authors provided input to editing for publication and accept responsibility to submit for publication.
