# Supplementary material for: ESPriT1: the effectiveness of laparoscopic treatment of isolated superficial peritoneal endometriosis for managing chronic pelvic pain in women: a randomised controlled feasibility trial
Source: Reprod Fertil. 2026 Jun 1;7(2):RAF260043. doi: 10.1530/RAF-26-0043 (PMC13232596; doi:10.1530/RAF-26-0043)
Supplement: Supplementary file 1 [file supplementary_materials.pdf]

Supplementary materials ESPriT1

Table 1 Participant demographics, obstetric outcomes and hormonal contraceptive use

Demographics	Consented but did not undergo surgery during the trial (n=31)	Underwent surgery but not eligible to be randomised (n=19)*	Randomised (n=7)	All consented participants (n=57)
Age (median, Q1-Q3)	27 (23-32)	30 (25-39)	27 (22-34)	27 (24-34)
Height[1] (cm) (median, Q1-Q3)	-	166 (159-170)	166 (157-168)	-
Weight[1] (kg) (median, Q1-Q3)	-	82 (60-94)	59 (52-86)	-
BMI[1](median, Q1-Q3)	-	30.1 (22.9-34.9)	21.2 (18.9-30.5)	-
Ethnicity				
White British	30 (97%)	18 (95%)	5 (71%)	53 (93%)
White Other	1 (3%)	1 (5%)	0	2 (4%)
Other	0	0	2 (29%)	2 (4%)
Education				
Secondary	7 (23%)	3 (16%)	1 (14%)	11 (19%)
Tertiary	24 (77%)	16 (84%)	6 (86%)	46 (81%)
Smoking within last 6 months				
No	21 (68%)	10 (53%)	5 (71%)	36 (63%)
Yes	10 (32%)	9 (47%)	2 (29%)	21 (37%)
Deprivation (n=33)				
1=most	2 (11%)	3 (33%)	0	5 (9%)
2	3 (17%)	2 (22%)	1 (17%)	6 (11%)
3	3 (17%)	4 (44%)	3 (50%)	10 (18%)
4	6 (33%)	0	1 (17%)	7 (12%)
5=least	4 (22%)	0	1 (17%)	5 (9%)
missing	13	10	1	24 (42%)
Evidence of adenomyosis				
No	29 (94%)	17 (100%)	7 (100%)	55 (96%)
Yes	2 (6%)	0	0	2 (4%)
Parity				
0	20 (65%)	10 (53%)	6 (86%)	36 (63%)
1	4 (13%)	5 (26%)	0	9 (16%)
2	5 (16%)	2 (11%)	0	7 (12%)
3	2 (6%)	2 (11%)	0	4 (7%)
4	0	0	1 (14%)	1 (2%)
Vaginal deliveries				
0	21 (68%)	11 (58%)	6 (86%)	38 (67%)
1	5 (16%)	6 (32%)	0	11 (19%)
2	3 (10%)	1 (5%)	0	4 (7%)
3	2 (6%)	1 (5%)	0	3 (5%)
4	0	0	1 (14%)	1 (2%)
Caesarean sections				
0	28 (90%)	18 (95%)	7 (100%)	53 (92%)
1	3 (10%)	0	0	3 (5%)
2	0	1 (5%)	0	1 (2%)
Investigation for infertility				
No	23 (74%)	12 (63%)	6 (86%)	41 (72%)
Yes	4 (13%)	1 (5%)	0	5 (9%)
Unknown	4 (13%)	6 (32%)	1 (14%)	11 (19%)
Planning future pregnancy				

No	2	7	2	11 (19%)
Yes	0	4	2	6 (11%)
Do not know	1	2	1	4 (7%)
Not answered	28	6	2	36 (63%)
Hormone use[1]				
**Any**	-	**7 (36.8%)**	**3 (42.9%)**	-
COCP	-	1 (5.2%)	0	-
LNG-IUS	-	2 (10.5%)	1 (14.3%)	-
Nexplanon	-	0	1 (14.3%)	-
Depo-Provera	-	1 (5.2%)	1 (14.3%)	-
POP	-	2 (10.5%)	0	-
GnRH with addback	-	2 (10.5%)	0	-

[1] only available in Randomisation table, only recorded for patients proceeding to protocol surgery (n=26).

[2]Reasons for non-randomisation were endometrioma (n=3), superficial peritoneal endometriosis but other ovarian cyst requiring surgical management (n=1), and no endometriosis observed (n=15). All diagnostic laparoscopies were performed (n=15) or directly supervised (n=10) by a Consultant Gynaecologist with the exception of one which was performed by an unsupervised senior trainee who specialised in benign gynaecological surgery (normal laparoscopy, not randomised).

Table *2* Surgical details of randomised participants

	**Diagnostic laparoscopy (n=4)**	**Surgical removal (n=3)**
Modality of removal:		
Ablation	N/A	2*
Excision	N/A	1
Additional procedures:		
Tubal dye test	0	1
LNG-IUS insertion	2	0

Ablation was performed by diathermy

Secondary outcomes

Table 3 Intraoperative and postoperative complications

	**Diagnostic laparoscopy only (n=4)**	**Surgical removal (n=3)**
Intraoperative complications during surgery?		
No	4	3
Yes	0	0
Overnight stay		
No	2	2
Yes	2	1
Any post-operative complications	2	3
No	2*	0
Yes		

*superficial wound infection. No participants required readmission to hospital

Table 4 Adverse events (AEs)

	**Diagnostic laparoscopy only (n=4)**	**Surgical removal (n=3)**
Number of participants reporting any AEs	2	2
Number of AEs per participant<br>0<br>1-2<br>3-4	 2<br>1<br>1	 1<br>1<br>1
Total number of AEs reported	4	5
Possibly related to procedure	3/4	1/5
Severity<br>Mild<br>Moderate	 4/4<br>0/4	 2/5<br>3/5
Description of AE	Low peak flow level, diagnosed with chest infection (mild, possibility related)	Nausea (mild, unrelated)
	Tonsillitis (mild, unrelated)	Vomiting (moderate, unrelated)
	Erythema around left laparoscopy wound site (mild, possibility related)	Pelvic pain (moderate, unrelated)
	Respiratory infection symptoms (mild, possibility related)	Haematuria (moderate, unrelated)
		Post op hypotension (mild, possibly)
Serious adverse events	0	0

Table 5 Proportion of complete EHP-30 questionnaires

**Timepoint**	**Diagnostic laparoscopy only (n=4)**	**Surgical removal (n=3)**
3 months	4	2
6 months	3	2
12 months	4	3

Table 6 Blinding

	**Diagnostic laparoscopy only (n=4)**	**Surgical removal (n=3)**
Number blinded at 12 months	4	3
Patient thinks endometriosis removed<br>No<br>Yes<br>Don't know	 2<br>1<br>1	 3<br>0<br>0

Table 7 Study acceptability to randomised participants

	Score (1=positive, 5=negative)				
	1	2	3	4	5
Overall has your trial experience been a positive or a negative one?	5	1	0	1	0
How was it to be approached for research?	5	1	1	0	0
How did you find the consent process?	7	0	0	0	0
How did you find the randomisation process?	4	1	2	0	0
How did you feel about the length of the questionnaires?	5	1	1	0	0
How comfortable did you feel completing the questionnaires?	7	0	0	0	0
How did you find the follow up process?	7	0	0	0	0
Did you feel supported during the study?	7	0	0	0	0

In those allocated to diagnostic laparoscopy alone, all 4 ranked overall acceptability as 1 ‘ most positive’. For those allocated to surgical removal the three participants ranked overall acceptability as 1, 2 and 4.

The efficacy outcomes of pelvic pain and quality of life, need for medical treatment (analgesics and ovarian suppression) are not reported here as only seven patients were randomised. These outcomes from ESPriT1 participants will be included in an IPD metanalysis with the ESPriT2 cohort. Requests for anonymised data will be considered by the ESPriT1 Trial Management Group (ETMT@ed.a.c.uk).
